# The mammalian protein MTCH1 can function as an insertase

**DOI:** 10.1242/jcs.263736

**Published:** 2025-08-15

**Authors:** Anna Roza Dimogkioka, Anni Elias, Doron Rapaport

**Affiliations:** Interfaculty Institute of Biochemistry, University of Tübingen, Tübingen 72076, Germany

**Keywords:** Insertase, MIM, Mitochondria, MTCH1, MTCH2, Outer membrane

## Abstract

The outer mitochondrial membrane (OMM) hosts a variety of proteins such as import machineries, enzymes, fission and fusion factors, and pore proteins. In *Saccharomyces cerevisiae*, the MIM complex, consisting of Mim1 and Mim2, mediates the insertion of α-helical proteins into the OMM. Until recently, it was unclear which proteins served this function in higher eukaryotes. Recent studies have identified MTCH2 as the insertase responsible for inserting α-helical proteins into the OMM in mammals. MTCH1 is a paralogue of MTCH2 but its general function and contribution to the biogenesis process are not clear. To better characterize MTCH1, we explored whether MTCH1 or MTCH2 could functionally replace Mim1 and/or Mim2 in yeast. Expression of MTCH1 and MTCH2 in yeast cells lacking Mim1, Mim2 or both revealed that MTCH1, but not MTCH2, could compensate for the growth defects upon deleting the MIM complex. Furthermore, MTCH1 could restore the biogenesis of MIM substrates, translocase of the outer membrane (TOM) complex stability and morphology of mitochondria. These findings indicate that MTCH1, by itself, has insertase activity and is a functional equivalent for the MIM complex, despite the absence of any evolutionary relation between the mammalian and yeast insertases.

## Teaser

**Summary:** The mammalian protein MTCH1, but not MTCH2, can functionally replace the yeast MIM complex in mediating membrane integration of mitochondrial outer membrane proteins.

## INTRODUCTION

Mitochondria play a central role in cellular metabolism, signalling and energy production. Despite possessing their own genome (mtDNA), mitochondria rely on nuclear-encoded proteins for their function. In fact, ∼1000 proteins in yeast and 1500 proteins in humans need to be synthesized in the cytosol before being transported to their specific mitochondrial sub-compartments (Schmidt et al., 2010; Wiedemann and Pfanner, 2017). As double-membrane organelles, mitochondria possess a unique architecture. The outer mitochondrial membrane (OMM) serves as a barrier to the cytosol while acting as a communication hub with the rest of the cell. The inner mitochondrial membrane (IMM) forms invaginations called cristae where oxidative phosphorylation occurs (Xian and Liou, 2021).

The OMM is home to a wide array of proteins, including those involved in protein import, enzymatic functions, mitochondrial fission and fusion, and pore formation (Papić et al., 2013). Dysfunction of these OMM proteins has been linked to human diseases like Alzheimer's, Parkinson's and mitochondrial encephalopathy (Bhatia et al., 2021; Henrich et al., 2023; Liu et al., 2021). OMM proteins exhibit distinct structural motifs, and are primarily categorized as either α-helical or β-barrel proteins. Although other mitochondrial protein import pathways have been well studied, the mechanism by which α-helical proteins are imported into the OMM has only been partially elucidated.

In *Saccharomyces cerevisiae*, the MIM complex, comprising Mim1 and Mim2, is responsible for the import of certain proteins containing one or more membrane-embedded α-helical segments (Becker et al., 2011; Waizenegger et al., 2005). Although the exact complex structure and stoichiometry remain unknown, it is speculated that Mim1 exists in the complex in excess to Mim2 (Dimmer et al., 2012; Popov-Čeleketić et al., 2008). Tom70 has been shown to be involved in the import of some MIM substrates, mainly acting as a receptor or docking site for precursor proteins such as Ugo1, Om14 and the mammalian protein PBR (also known as TSPO) (Becker et al., 2011; Papić et al., 2013; Zhou et al., 2022). Initially, the proteins responsible for the membrane insertion of α-helical proteins in other eukaryotes remained unclear.

**Fig. 1.**
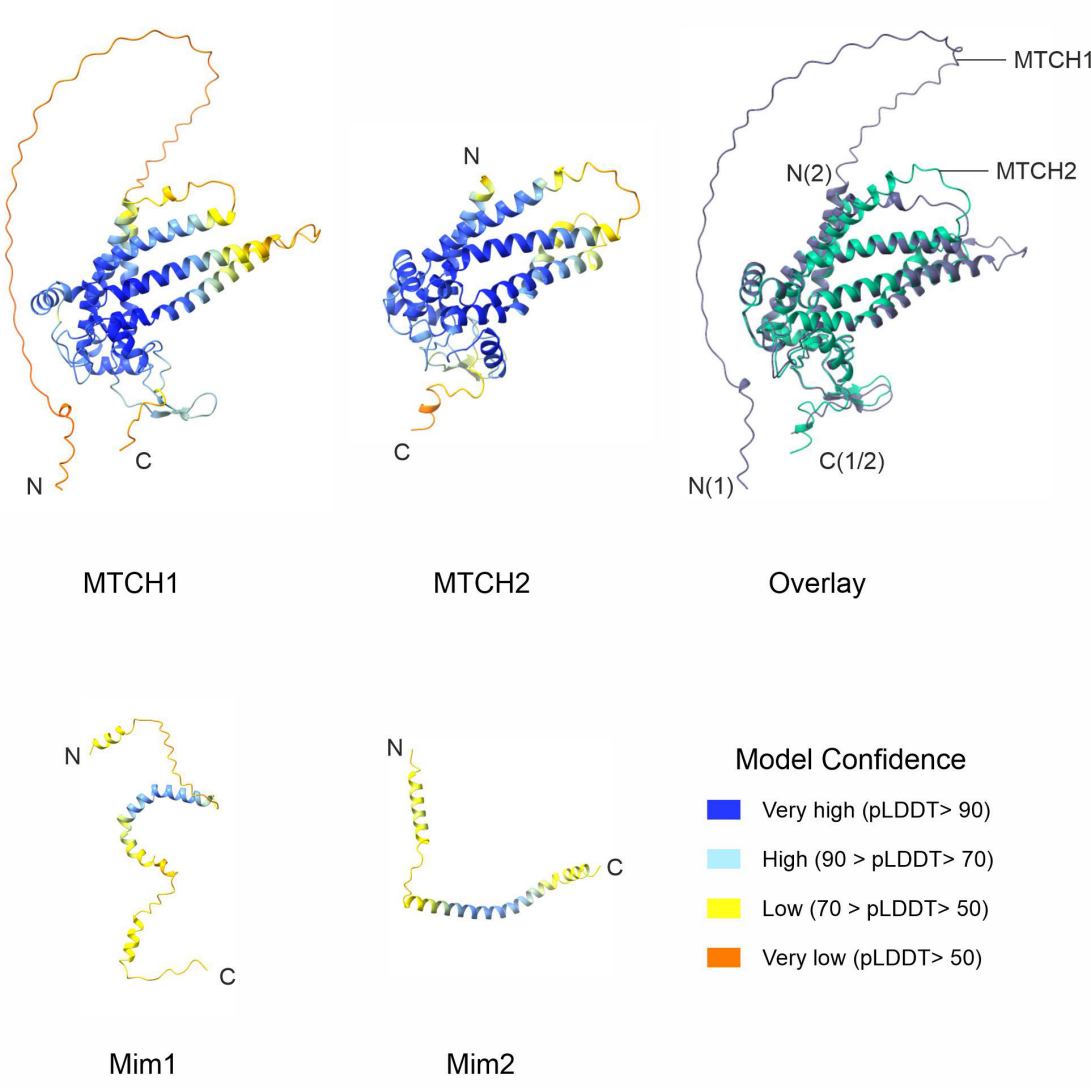
**MTCH1 and MTCH2 are multi-span proteins that share structural similarity.** Structural prediction of MTCH1 (UniProt: Q9NZJ7), MTCH2 (UniProt: Q9Y6C9), Mim1 (Uniprot: Q08176) and Mim2 (Uniprot: Q3E798) adapted from AlphaFold. Overlay of MTCH1 and MTCH2 structural predictions is also included. MTCH1 and MTCH2 structural predictions were aligned using Chimera X. Bottom right shows model confidence table corresponding to the AlphaFold structures. Protein structural models were computed using AlphaFold (https://alphafold.ebi.ac.uk/) using the amino acid sequences of the relevant proteins as input.

In 2018, Vitali et al. identified pATOM36 as the functional equivalent of the MIM complex in *Trypanosoma brucei* (Vitali et al., 2018). Later, in 2022, Guna et al. demonstrated that MTCH2 facilitates the import of a subset of α-helical mammalian OMM proteins (Guna et al., 2022). MTCH2 and its understudied paralogue MTCH1 are members of the SLC25 membrane carrier family. Unlike most SLC25 proteins, which localize to the IMM, MTCH1 and MTCH2 are situated in the OMM (Ruprecht and Kunji, 2020). Both proteins carry six putative transmembrane α-helices with MTCH2 having 303 amino acid residues whereas MTCH1 harbours a total of 389 amino acids. Most of this difference is due to an extended N-terminal domain of MTCH1, which is predicted by AlphaFold to be unstructured (Fig. 1). Of note, MTCH1 has been recently identified as a potential anti-ferroptosis factor in cervical cancer (Wang et al., 2023). However, so far, much remained unknown about MTCH1, and although its downregulation together with reduced levels of MTCH2 resulted in compromised insertion of some OMM proteins (Guna et al., 2022), its single knockdown did not affect biogenesis of OMM proteins (Muthukumar et al., 2024; Wang et al., 2023). MTCH2, on the other hand, has been extensively studied, with suggested roles in lipid homeostasis, mitochondria fusion and apoptosis (Labbé et al., 2021; Rottiers et al., 2017; Zaltsman et al., 2010), and more recently lipid scrambling (Bartoš et al., 2024; Li et al., 2024). Employing coarse-grained molecular dynamics simulations, Li et al. (2024) concluded that MTCH1 also can have scrambling activity.

Notably, MTCH1 and MTCH2 share no sequence similarity with Mim1, Mim2 or pATOM36. Although certain SLC25 carrier family proteins are conserved between yeast and mammals, MTCH1 and MTCH2 are not. Given these differences, we sought to investigate whether MTCH1 and/or MTCH2 could functionally complement the absence of Mim1, Mim2 or both in yeast. To that end, we expressed the former proteins in yeast strains mutated for Mim components. Remarkably, MTCH1, but not MTCH2, was able to complement the growth defects associated with the loss of Mim1 and Mim2. Furthermore, MTCH1 restored MIM substrate biogenesis, translocase of the outer membrane (TOM) complex stability and mitochondrial morphology in the MIM-mutated cells. These findings suggest that, despite the absence of evolutionary conservation between these mammalian and yeast proteins, MTCH1 possesses inherent insertase activity and functions as a homologue of the MIM complex.

## RESULTS

### MTCH1 complements the growth defects in cells lacking Mim1, Mim2 or both

Given that both MTCH1 and MTCH2 have been implicated in the biogenesis of α-helical OMM proteins (Guna et al., 2022), we sought to investigate their effects when expressed in *S. cerevisiae*. Structurally, both MTCH1 and MTCH2 possess six putative transmembrane α-helices; however, MTCH1 is larger due to a long unstructured N-terminal domain (Fig. 1). Structural analysis via AlphaFold (Jumper et al., 2021) predicts tighter α-helices in MTCH2 compared to MTCH1, a difference confirmed by computational structural alignment (Fig. 1). As the structure of the mitochondrial import (MIM) complex remains unresolved, a direct structural comparison with MTCH1 and MTCH2 was not possible. Furthermore, structural predictions for the individual components of the MIM complex, Mim1 and Mim2, are of low confidence and thus not suitable for meaningful comparative analysis (Fig. 1).

To test whether MTCH1 and/or MTCH2 could functionally substitute for the MIM complex *in vivo*, we wanted to express these proteins in wild-type (WT) cells, or in the single deletion strains *mim1Δ* and *mim2Δ*, as well as in the double deletion (*mim1/2ΔΔ*). To that goal, the sequences of MTCH1 and MTCH2 were codon optimized for yeast expression and C-terminally tagged with HA to facilitate detection. As controls, the empty yeast expression plasmid pRS426 and pRS426 plasmid encoding either Mim1 or Mim2 were transformed into the relevant strains. The growth of these strains was monitored at 30°C and 37°C using both solid and liquid media, with glucose or galactose as the carbon source. These specific conditions were selected as they are known to accentuate the growth defects associated with the loss of Mim proteins (Dimmer et al., 2012; Vitali et al., 2018).

Initial experiments with C-terminally HA-tagged MTCH1 and MTCH2 revealed partial growth complementation of the *mim1Δ*, *mim2Δ* and *mim1/2ΔΔ* strains by MTCH1 (Fig. S1). Surprisingly, MTCH2, which had been identified as a key insertase for α-helical OMM proteins (Guna et al., 2022), not only failed to rescue the growth defect but also had a strong inhibitory effect on the growth of all strains, including WT cells (Fig. S1).

**Fig. 2.**
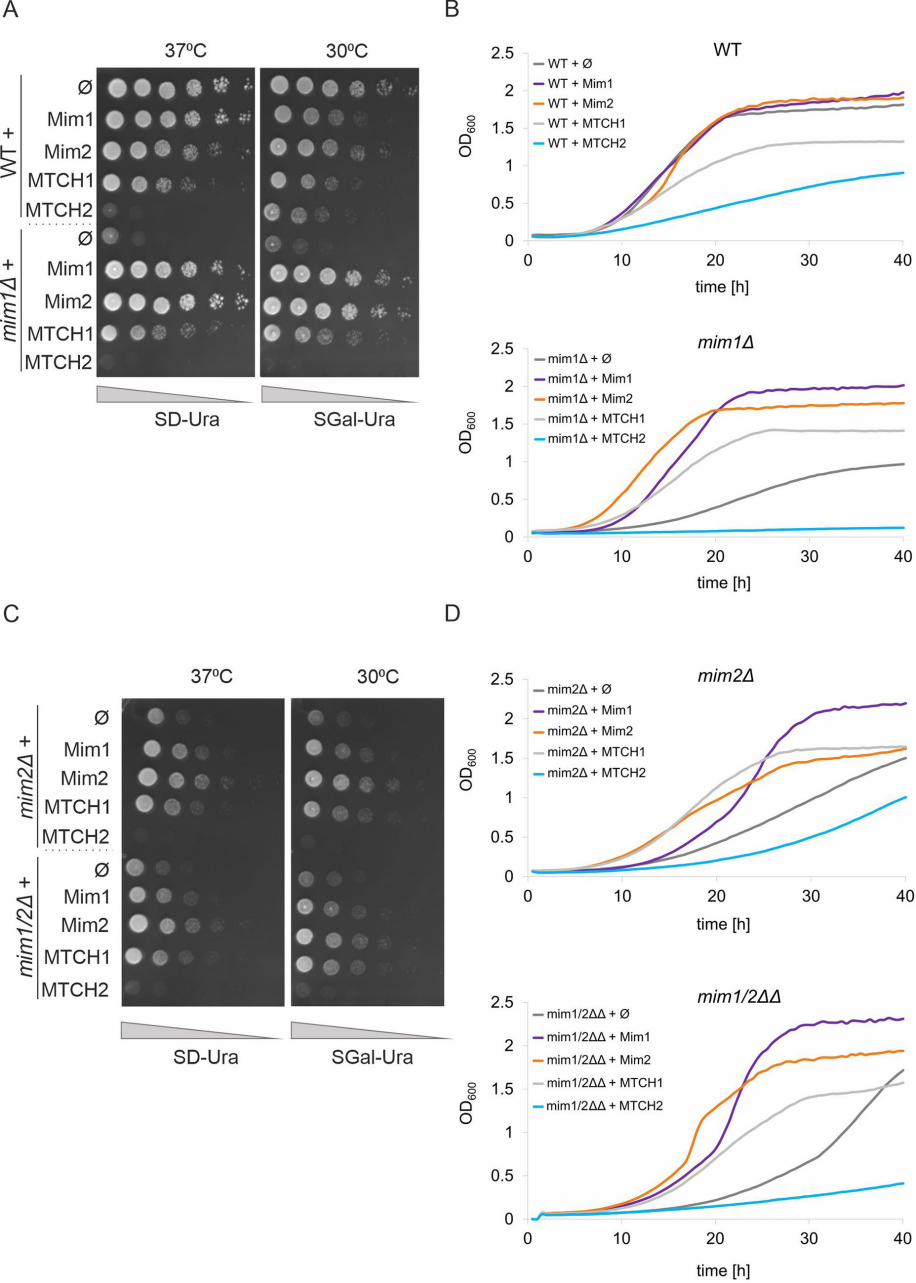
**MTCH1 can complement the growth defects in cells lacking Mim1, Mim2 or both.** (A,C) The growth of the indicated strains was monitored by drop dilution assay on solid synthetic medium containing either glucose (SD) or galactose (SGal) at either 30°C or 37°C. The strains were transformed with an empty vector (Ø) or vector encoding the indicated protein. Plates were incubated for 2 days at the indicated temperature before pictures were taken. (B,D) The growth of the indicated strains at 37°C in liquid glucose-containing medium was observed for 40 h using OD_600_ measurements. At the beginning of the measurements (time 0), the strains were diluted to an OD_600_ of 0.1. Data in this figure is representative of at least three repeats.

Considering these unexpected findings, we next wondered whether the HA tag might be responsible for this ‘dominant negative’ phenotype of MTCH2 expression. To test this, we expressed both MTCH1 and MTCH2 without the HA tag. Notably, untagged MTCH1 complemented the absence of Mim1 and Mim2 more effectively than the tagged version. In assays with both solid and liquid media, MTCH1 significantly rescued the growth defects, reaching complementation levels comparable but not equal to those of Mim1 and Mim2 (Fig. 2). Remarkably, MTCH1 alone was able to restore growth in the absence of both Mim proteins, indicating that its activity is independent of pre-existing Mim1 or Mim2. Although a very slight negative effect on the growth of WT cells was observed with MTCH1 expression, its expression was beneficial in all other strains (Fig. 2).

In contrast, MTCH2 exhibited a negative effect on cell growth, even without the HA tag. The most severe growth defect was observed when MTCH2 was expressed in the *mim1Δ* strain (Fig. 2B), with only slight improvements in the *mim2Δ* and *mim1/2ΔΔ* strains (Fig. 2D). In summary, MTCH1 consistently rescued the growth of cells lacking Mim1, Mim2 or both, in both solid and liquid media.

**Fig. 3.**
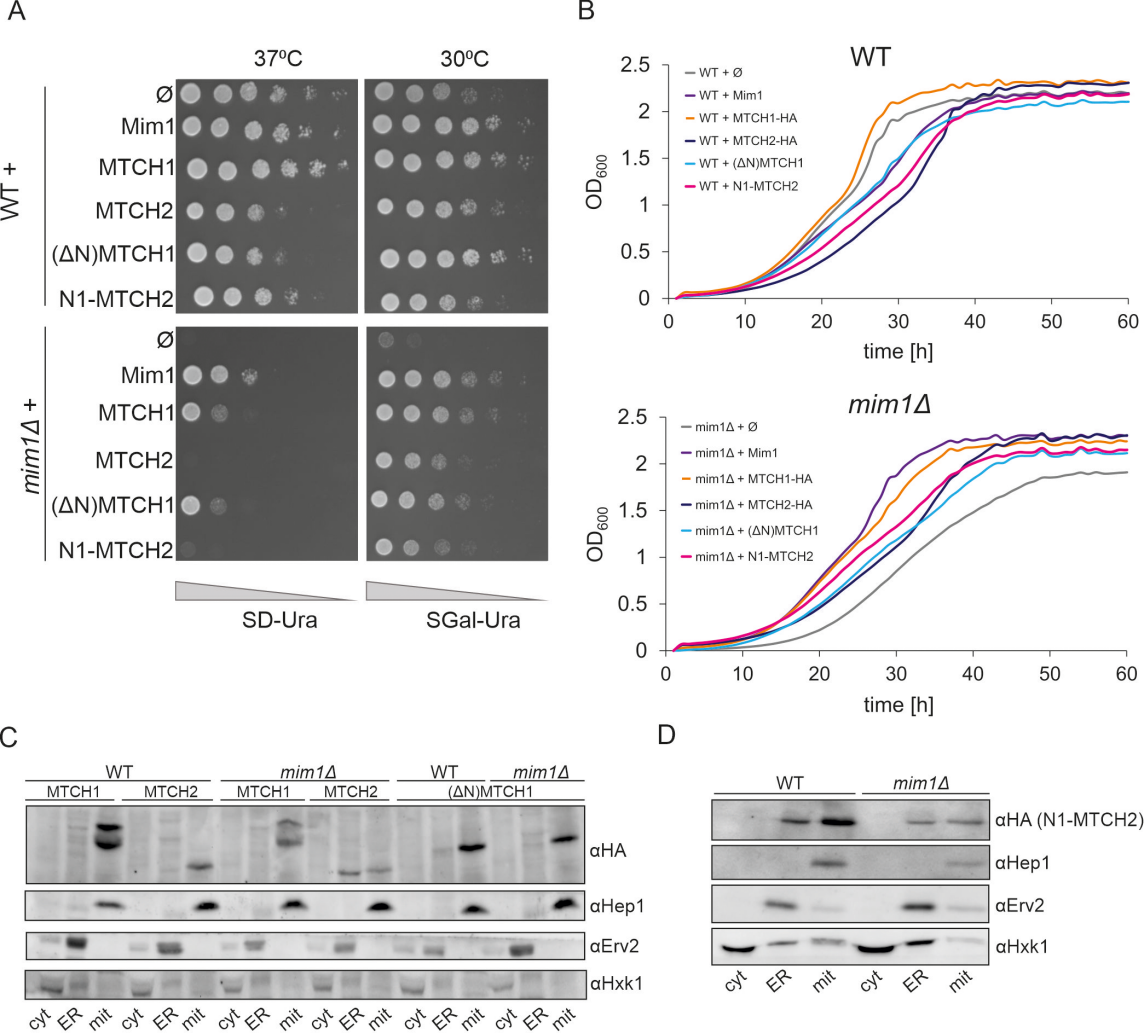
**The N-terminal segment of MTCH1 contributes to its capacity to complement the absence of Mim1.** (A) The growth of the indicated strains was monitored by drop dilution assay on solid synthetic medium containing either glucose (SD) or galactose (SGal) at either 30°C or 37°C. The strains were transformed with an empty vector (Ø) or vector encoding the indicated protein. Plates were incubated for a few days at the indicated temperature before pictures were taken. (B) The growth of the indicated strains at 37°C in liquid glucose-containing medium was monitored for 60 h using OD_600_ measurements. At the beginning of the measurements (time 0), the strains were diluted to an OD_600_ of 0.1. (C,D) Fractions corresponding to cytosol (cyt), ER and mitochondria (mit) were isolated from the indicated cells expressing the depicted HA-tagged proteins. The samples were analysed by SDS-PAGE and immunodecorated with the indicated antibodies. Hep1 is a mitochondrial protein; Erv2 is an ER protein; hexokinase (Hxk1) is a cytosolic marker. Data in this figure is representative of at least three repeats.

One potential explanation for the observed toxicity of MTCH2 might lie in the expression levels of the proteins. The initial experiments used the multi-copy yeast expression plasmid pRS426, which contains a TPI promoter. High expression levels of MTCH2 from this vector might have led to cellular toxicity. This possibility is supported by observations in WT cells (Fig. 2A), where overexpression of both Mim1 and MTCH1 also resulted in a mild growth defect. To test this hypothesis, Mim1, MTCH1–HA and MTCH2–HA were subcloned into the centromeric plasmid pYX142, which also uses the TPI promoter but has a lower copy number. The resulting strains were analysed using both liquid and solid media growth assays, as shown in Fig. 3. In WT cells, expression of MTCH2–HA from the pYX142 plasmid resulted in a less pronounced growth defect compared to expression from the high-copy vector (Fig. 3A). This improvement is more evident in the liquid growth assay (Fig. 3B), where strains expressing MTCH2 showed a growth rate more comparable to those expressing Mim1 and MTCH1–HA.

Given this improved growth in WT cells, we next tested whether MTCH2–HA could better complement the absence of Mim1 under the lower expression conditions. As shown in Fig. 3B, the *mim1Δ* strain expressing MTCH2–HA grew somewhat better than the deletion strain carrying an empty vector. Although its growth did not reach the level of the cells expressing Mim1 or MTCH1, this still represents a notable improvement over the near complete growth inhibition observed with MTCH2 expression from the high-copy plasmid. These results indicate that the cellular toxicity of MTCH2 is dose dependent and although lower expression improves viability, MTCH2–HA is still unable to complement the absence of Mim1 as effectively as MTCH1–HA or Mim1 itself. It is also interesting that MTCH2 levels specifically are so toxic to the cell, given that MTCH1 only had a minor negative growth effect in WT cells when expressed at very high levels and could complement the absence of Mim1 irrespective of the level of expression.

### The N-terminal region of MTCH1 might have a role in insertase function

As the most prominent structural difference between MTCH1 and MTCH2 lies in the N-terminal region, we hypothesized that the unstructured N-terminus of MTCH1 might contribute to its insertase function. To explore this, we created a truncated version of MTCH1 lacking the first 78 amino acid residues [(ΔN)MTCH1] with a C-terminal HA tag, cloned into the pYX142 vector for moderate expression. WT cells expressing (ΔN)MTCH1 showed growth patterns similar to those harbouring full-length MTCH1 at 30°C in galactose-containing medium but exhibited a slower growth rate at 37°C on glucose (Fig. 3A). Liquid growth assays revealed that cells expressing (ΔN)MTCH1 grew slower than those containing MTCH1. In *mim1Δ* cells, (ΔN)MTCH1 still complemented the growth phenotype on solid medium to levels comparable to full-length MTCH1, but the liquid growth curves more resembled those of MTCH2–HA (Fig. 3A,B). These findings suggest that the N-terminal region of MTCH1 enhances its complementation efficiency in cells lacking Mim1.

Next, we wanted to test whether adding the MTCH1 N-terminal segment to MTCH2 could improve its ability to complement the deletion of Mim1. We created the chimeric construct N1-MTCH2 by fusing the first 78 amino acids of MTCH1 to the N-terminus of MTCH2 and adding a C-terminal HA tag. WT cells expressing N1-MTCH2 displayed a growth phenotype similar to those with MTCH2, with a slight additional growth defect at 30°C, as reflected in the respective growth curve (Fig. 3A,B). In *mim1Δ* cells, N1-MTCH2 performed slightly better than MTCH2 on solid media, with more significant improvement in liquid growth assays (Fig. 3A,B). Whereas the chimeric protein did not reach the complementation efficiency of MTCH1 or Mim1, these results suggest that the MTCH1 N-terminus modestly enhances MTCH2 function in the absence of Mim1.

To better understand this effect, we investigated the subcellular localization of the various constructs. Using a low-copy plasmid, we found that MTCH2-HA localizes primarily to mitochondria in WT cells, comparable to the control mitochondrial protein Hep1 (Fig. 3C). In contrast, in *mim1Δ* cells, most of the MTCH2–HA signal was detected in the endoplasmic reticulum (ER), with a smaller fraction localized to mitochondria. (ΔN)MTCH1 localized exclusively to mitochondria in both WT and *mim1Δ* cells, indicating that deletion of the N-terminus does not impair mitochondrial targeting for MTCH1. By contrast, the addition of the MTCH1 N-terminal region to MTCH2 did affect its localization; in WT cells the majority of the protein was found in mitochondria, but a small population was still localized to the ER (Fig. 3D). In the deletion strain, however, the protein seems to be more evenly distributed between the ER and mitochondria compared to MTCH2, which was found mainly in the ER (Fig. 3D). This redistribution might explain the improved growth observed with N1-MTCH2 in *mim1Δ* cells. Taken together, these findings suggest that the N-terminal segment of MTCH1 plays a certain role in determining its mitochondrial localization and functional capacity.

### MTCH1 restores the biogenesis of MIM complex substrate and reconstitutes TOM complex assembly

**Fig. 4.**
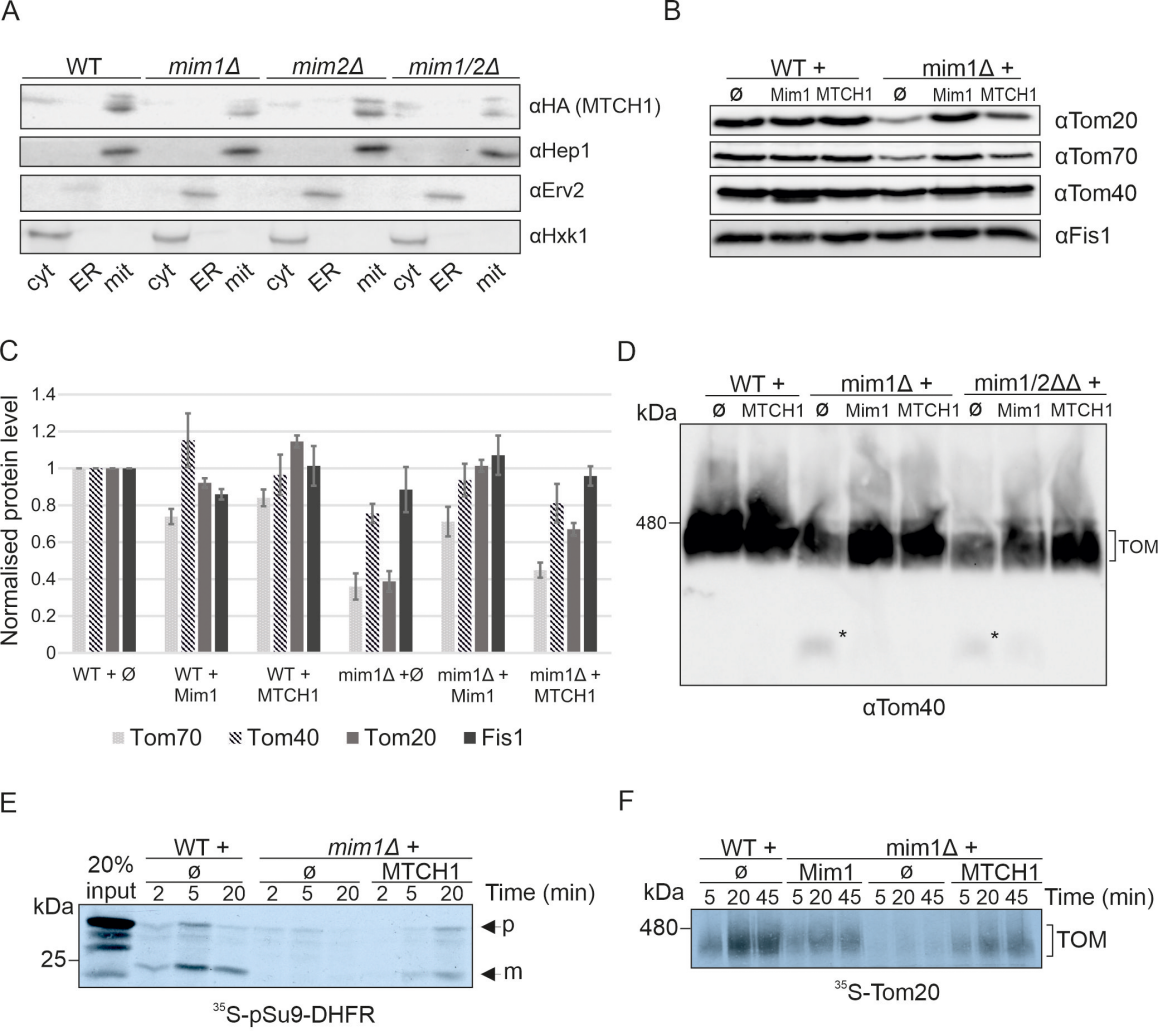
**MTCH1 rescues the reduced steady state levels and assembly defects in cells deleted for Mim components.** (A) Subcellular fractionation of WT, *mim1Δ*, *mim2Δ* and *mim1/2ΔΔ* strains expressing MTCH1-HA. Cytosol (cyt), ER and mitochondrial (mit) fractions were obtained using differential centrifugation steps. The samples were analysed by SDS-PAGE and immunodecorated with the indicated antibodies. Hep1 is a mitochondrial protein; Erv2 is an ER protein; hexokinase (Hxk1) is a cytosolic marker. (B) Mitochondria (100 µg) isolated from the indicated strains were analysed by SDS-PAGE and immunodecoration with antibodies against the indicated mitochondrial proteins. (C) The bands corresponding to the indicated proteins from three independent experiments as the one shown in B were quantified and normalized to the intensities of the Ponceau S staining. The value in the WT cells with empty plasmid was set as 100%. The bar diagram shows the mean±s.d. of three independent experiments. (D) Isolated mitochondria from the indicated strains were solubilized with 1% digitonin. Samples were analysed by BN-PAGE and immunodecorated with antibodies against Tom40. The bands representing the assembled TOM complex are indicated. Asterisk (*) indicates dissociated form of the TOM complex. (E) Radiolabelled pSu9-DHFR was synthesized in a cell-free system and imported into mitochondria isolated from the indicated cells. After import for the indicated times, proteinase K was added. Samples were analysed by SDS-PAGE and autoradiography. The precursor (p) and mature (m) forms of the protein are indicated. (F) Radiolabelled Tom20 was imported for the specified time periods into mitochondria isolated from the indicated cells. Samples were analysed via BN-PAGE and autoradiography. The band corresponding to the assembled TOM complex is indicated. Data in A, D–F is representative of three repeats.

Although MTCH1 successfully complemented the growth defects in strains lacking Mim1 and/or Mim2 even upon high expression levels, it was unclear whether MTCH1 under these conditions localizes to mitochondria and compensates for other defects associated with the loss of Mim proteins. To confirm mitochondrial localization, we performed subcellular fractionation on cells highly expressing MTCH1–HA, separating cytosolic, endoplasmic reticulum (ER) and mitochondrial fractions. Hexokinase1 served as a cytosolic marker, Erv2 for the ER, and Hep1 for the mitochondria. As shown in Fig. 4A, MTCH1–HA was found in the mitochondrial fraction alongside Hep1, with small traces in the cytosolic fraction and no detectable presence in the ER fractions, indicating that MTCH1 localizes to yeast mitochondria even in the absence of Mim1 and/or Mim2. Owing to the low complementation capacity of all constructs beside MTCH1, we decided to continue with a complete analysis only of the strain expressing this protein.

Deletion of Mim1 and Mim2 also leads to reduced levels of several MIM complex substrates, particularly α-helical OMM proteins, such as Tom20 and Tom70. We next examined whether MTCH1 could restore the steady-state levels of these α-helical OMM proteins in the Mim deletion strains. Additionally, we analysed Tom40, a β-barrel OMM protein, whose levels are indirectly affected by the absence of Mim1, and Fis1, which is imported independently of the MIM complex (Vitali et al., 2020), to ensure that the MTCH1 effects were specific to α-helical OMM proteins. WT and *mim1Δ* deletion strains expressing either empty pRS426 plasmid (Ø) or plasmid-encoded MTCH1 were grown in synthetic medium with lactic acid as the carbon source to promote mitochondrial biogenesis. Cells were then lysed, mitochondria were isolated and their proteins were analysed via SDS-PAGE followed by immunodetection.

The results demonstrated that MTCH1 restored the levels of Tom70 and Tom20 in the deletion strain to levels comparable to those observed in Mim1-expressing cells. Importantly, the expression of MTCH1 in WT cells altered only slightly, if at all, the abundance of these proteins (Fig. 4B,C), indicating that MTCH1 effects are specific to Mim-deficient strains and its activity is not beneficial in the presence of a functional MIM complex.

The loss of Mim1 and Mim2 also disrupts the assembly of the TOM complex, leading to the accumulation of assembly intermediates and reduced levels of the mature TOM complex (Dimmer et al., 2012). This defect arises because several TOM components (like Tom20 and Tom70) are α-helical proteins, whose biogenesis is impaired in the absence of the MIM complex. To determine whether MTCH1 expression could correct these assembly defects, we performed blue native (BN)-PAGE analysis on mitochondria isolated from WT and Mim deletion strains transformed with either empty pRS426 plasmid or a plasmid encoding MTCH1. As shown in Fig. 4D, MTCH1 expression alleviated the TOM complex assembly defects observed in *mim1Δ* and *mim1/2ΔΔ* strains. Specifically, MTCH1 reduced the accumulation of TOM complex intermediates (∼100 kDa, indicated by an asterisk) and promoted the formation of the assembled TOM complex (∼440 kDa) to levels comparable to those in WT mitochondria. These findings indicate that MTCH1 can not only restore the levels of MIM substrates but also improve TOM complex assembly in Mim-deficient strains.

Our findings demonstrate that MTCH1 can rescue growth defects, restore steady-state levels of α-helical mitochondrial outer membrane (MOM) proteins and reconstitute TOM complex assembly in *mim1Δ* strains. To further investigate the involvement of MTCH1 in the biogenesis of MIM complex substrates, we employed a cell-free translation system to synthesize radiolabelled substrate proteins. These labelled substrates were incubated at 25°C for various time periods with mitochondria isolated from either WT or *mim1Δ* cells transformed with plasmids encoding MIM1 and MTCH1.

Although not a specific substrate of the MIM complex, pSu9-DHFR import serves as a general indicator of mitochondrial protein import efficacy, which is known to be (indirectly) compromised when the MIM complex is mutated (Mnaimneh et al., 2004). Thus, we examined the import of pSu9-DHFR, a model fusion protein comprising the pre-sequence of F_0_-ATPase subunit 9 (pSu9) and mouse dihydrofolate reductase (DHFR). During import, pSu9-DHFR is cleaved by the matrix processing peptidase (MPP) producing a mature form (Fig. 4E) (Pfanner et al., 1987). In *mim1Δ* mitochondria, we observed a marked reduction in pSu9-DHFR import, indicating a general defect in mitochondrial protein import. The expression of MTCH1 in *mim1Δ* cells improved pSu9-DHFR import (Fig. 4E).

Finally, we monitored the biogenesis of Tom20, a signal-anchored protein that is embedded in the OMM. Tom20 is known to be a peripheral component of the TOM complex and to heavily rely on the MIM complex for its biogenesis (Hulett et al., 2008; Popov-Čeleketić et al., 2008). Therefore, to monitor the import of newly synthesized radiolabelled Tom20 molecules into isolated organelles, Tom20 association with the TOM complex was assessed using BN-PAGE. To account for the weak association of Tom20 with the rest of the TOM complex, gentle solubilization of mitochondria was applied [digitonin:protein ratio of 1:1 (w/w)]. This BN-PAGE analysis revealed that Tom20 assembly with the TOM complex was impaired in *mim1Δ* mitochondria, with slower import kinetics compared to that seen in control organelles. Importantly, MTCH1 expression in *mim1Δ* cells improved the rate of Tom20 integration into the TOM complex, similar to the effect observed upon re-introducing Mim1 (Fig. 4F).

### MTCH1 improves mitochondrial morphology in strains lacking Mim1

To further confirm that MTCH1 functions as a homologue of the MIM complex, we examined the overall mitochondrial network morphology. Cells lacking Mim1, Mim2 or both exhibit characteristic mitochondrial defects, including fragmentation and punctate structures, in contrast to the tubular mitochondrial network seen in WT cells (Dimmer et al., 2012). To visualize the mitochondrial network, we expressed a fusion protein consisting of GFP linked to mitochondrial targeting presequence pSu9 (mt-GFP). We expressed pSu9-GFP in *mim1Δ* cells harbouring an empty pRS426 plasmid, or a plasmid encoding either Mim1 or MTCH1.

**Fig. 5.**
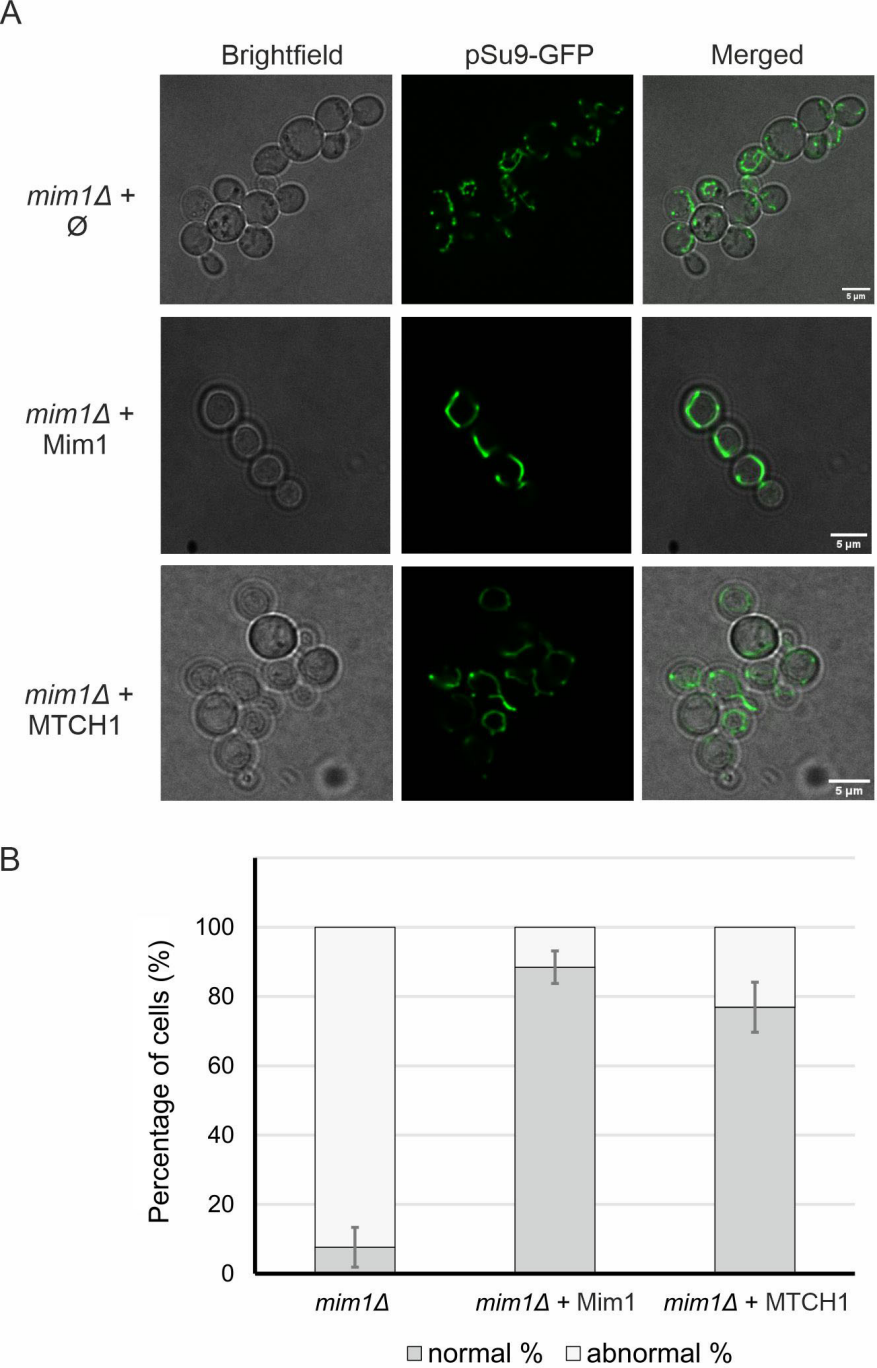
**MTCH1 can complement the mitochondrial morphology defect of *mim1Δ* cells.** (A) *mim1Δ* cells harbouring mitochondria-targeted GFP (mtGFP) were transformed with either an empty plasmid (Ø) as a control (upper panels) or a plasmid encoding either MIM1 or MTCH1 (middle and lower panels, respectively). Cells were analysed by fluorescence microscopy and representative images of the predominant morphology for each strain are shown. Scale bar: 5 µm. (B) Quantification of the cells described in A where cells with either normal or abnormal mitochondrial morphology were counted. Mean±s.d. of three independent experiments with at least *n*=100 cells in each experiment are shown.

As expected, *mim1Δ* cells predominantly displayed a fragmented mitochondrial network with the formation of punctate structures (Fig. 5A). When Mim1 expression was restored, the cells regained a tubular mitochondrial network consistent with the morphology observed in healthy mitochondria (Fig. 5A,B). Strikingly, cells expressing MTCH1 also exhibited a largely restored tubular mitochondrial network with only ∼20% of the cells still displaying abnormal mitochondrial morphology (Fig. 5A,B). This major restoration suggests that MTCH1 can support also the recovery of mitochondrial structure.

## DISCUSSION

MTCH1 has previously been suggested to be involved in the import of α-helical OMM proteins in mammalian cells; however, clear evidence for such a function was missing. Our study demonstrates that MTCH1 alone can functionally complement the absence of Mim1, Mim2 or both in *S. cerevisiae* and rescue the import defects in mitochondria lacking a functional MIM insertase. Although MTCH1 and MTCH2 share structural similarities and are members of the same protein family, their effects in yeast are markedly different. Specifically, in contrast to the positive effects of MTCH1, high levels of MTCH2 caused an inhibitory effect on the growth of cells. It might be that the high amounts of MTCH2 at the OMM of WT cells generally disturb the integrity of the OMM and thus compromise mitochondrial function. The inhibitory effect of MTCH2 was significantly improved by lowering the expression levels of the protein; however, the reduced amounts only slightly improved its ability to complement the absence of Mim1. Fusing the N-terminal region of MTCH1 to MTCH2 helped improve its effect on growth but still not to the level of Mim1 and MTCH1. In addition, the N-terminal region helped redistribute MTCH2 to mitochondria from the ER, where it was mostly found in *mim1Δ* cells.

The localization of MTCH2 to the ER in cells lacking Mim1 might be interfering with the import machinery on the ER surface or disturbing the lipid bilayer, causing ER disfunction and leading to impaired growth. However, even upon redistribution to the mitochondria caused by the addition of the MTCH1 N-terminal region, MTCH2 did not manage to complement the absence of Mim1. The ability of MTCH1 to functionally replace the MIM complex in yeast mitochondria suggests that MTCH1 possesses distinct properties that make it more adaptable to yeast systems. The N-terminal domain that is absent in MTCH2 has a partial effect but is not enough to explain the difference in complementation between the proteins. Structural prediction analysis revealed that the α-helices of MTCH2 are more tightly packed compared to those of MTCH1, potentially suggesting that MTCH1 has a conformation better suited for integration into the yeast mitochondrial membrane. Finally, although the two proteins share some sequence similarity, it could be that MTCH1 has a specific motif or region that is crucial for substrate recognition or docking, enabling MTCH1 to act as an efficient insertase in yeast.

Moreover, although structural data for the MIM complex remains unavailable, future studies comparing the structure of MTCH1 to that of the MIM complex could reveal some evolutionary conservation. It is possible that MTCH1 has more structural similarity to the MIM complex than MTCH2, allowing it to perform the same roles in protein import. MTCH2, on the other hand, might have evolved later to fulfil more specialized or complementary functions in complex tissues and cells of mammalian organisms. The lack of sequence and apparent structural similarity between Mim subunits and MTCH1, despite their related functions, is in line with the previous identification of the protein pATOM36 as the functional equivalent of the MIM complex in *T. brucei* (Vitali et al., 2018). Also, pATOM36 and the Mim components do not share any sequence and structural resemblance. Hence, it seems that the different insertases of the OMM are the result of convergent evolution.

Our novel findings open avenues for further exploration into the evolutionary significance of functional conservation in mitochondrial biogenesis. To provide deeper insights into the functional redundancy and evolutionary history of mitochondrial insertases across species, future studies might test the reciprocal complementation, where both components of the MIM complex (Mim1 and Mim2) are co-expressed in human cell lines lacking MTCH1 and/or MTCH2. It is hard to predict whether such co-expression will restore function in a manner similar to our findings in yeast.

In summary, our current findings provide clear evidence for the capacity of the mammalian protein MTCH1 to act as an insertase for mitochondrial outer membrane proteins.

## MATERIALS AND METHODS

### Yeast strains and growth conditions

Yeast strains of the genetic background W303α were used in this study (Table S1). Yeast cells were grown at either 30°C or 37°C in synthetic medium lacking uracil (1.9 g yeast nitrogen base without ammonium sulphate, 5 g ammonium sulphate, 55 mg adenine sulphate) supplemented with a 100× stock of amino acids and either glucose (2%) or galactose (2%) (SD-Ura or SGal-Ura, respectively). For mitochondrial isolation, cells were grown in S-uracil medium supplemented with lactate (2%). Yeast strains were transformed with the desired plasmid(s) using the lithium acetate method.

### Recombinant DNA methods

For better expression in yeast cells, the DNA encoding sequences of both MTCH1 and MTCH2 were optimized for yeast codon usage utilizing the Eurofins Genomics codon optimization tool. Both genes were cloned into the pRS426 and pYX142 vectors for yeast expression purposes with EcoRI and BamHI as the restriction sites. A detailed lists of primers and plasmids are included in Tables S2 and S3, respectively.

### Cell growth assays

For growth assay on solid medium (drop dilution assays), yeast strains were grown in SD-ura medium to an OD_600_=1. The cells were then serially diluted five times in fivefold increments. Next, 5 µl from each dilution was placed onto the solid medium and the plates were incubated at 30°C or 37°C for several days. For the liquid growth curve assays, yeast strains were grown to an OD_600=_1 and diluted to OD_600_=0.1. Triplicates of each strain were loaded onto a 96-well Microtest plate and the OD_600_ was measured every 30 min for up to 60 h using the SPECTROstar Nano plate reader. Analysis of the growth curves was performed using the SPECTROstar Nano MARS software.

### Biochemical methods

#### Isolation of mitochondria

Yeast cells were grown in liquid medium (volume of 2–6 l) to logarithmic phase. The cells were harvested (3000 ***g***, 5 min, RT), resuspended in DTT buffer (100 mM Tris, 10 mM DTT) and incubated at 30°C for 15 min. Cells were harvested (2000 ***g***, 5 min, RT), washed once with spheroplasting buffer (1.2 M Sorbitol, 20 mM potassium phosphate, pH 7.2), harvested again and resuspended in spheroplasting buffer with Zymolyase (5 mg/g of cells) and incubated at 30°C for 1 h.

Further steps were carried out on ice. Spheroplasts were homogenized in homogenization buffer [0.6 M Sorbitol, 10 mM Tris-HCl pH 7.4, 1 mM EDTA, 0.2% fatty acid-free BSA with 2 mM phenylmethylsulfonyl fluoride (PMSF)] using a dounce homogenizer to obtain a cell lysate. Cell debris and nuclei were removed by two clarifying spins (2000 ***g***, 10 min, 4°C). The supernatant (cytosol+organelles) was centrifuged (18,000 ***g***, 15 min, 4°C) to pellet crude mitochondria. The resulting post-mitochondrial supernatant (PMS) consisted of ER/microsomal and cytosolic fractions. The crude mitochondria were washed twice with SEM buffer (250 mM sucrose, 1 mM EDTA, 10 mM MOPS, pH 7.2) containing 2 mM PMSF and were pelleted again (18,000 ***g***, 15 min, 4°C).

#### Subcellular fractionation

All the steps were carried out at 4°C. Whole-cell lysate and crude mitochondria were obtained as described above. To further purify mitochondria from potential contaminants, the mitochondrial fraction was layered on a Percoll gradient (25% Percoll, 2 M sucrose, 100 mM MOPS/KOH pH 7.2, 100 mM EDTA, 200 mM PMSF) and centrifuged (80,000 ***g***, 45 min, 4°C). Highly pure mitochondria were found as a brownish layer close to the bottom of the tube and was removed carefully with a Pasteur pipette. The mitochondria were washed several times with SEM buffer containing 2 mM PMSF and pelleted again (18,000 ***g***, 15 min, 4°C).

To isolate ER/microsomal and cytosolic fractions, 20 ml of PMS were clarified (18,000 ***g****,* 15 min, 4°C) and centrifuged (200,000 ***g***, 1 h, 4°C). The supernatant contained the cytosolic fraction. The brownish sticky pellet (consisting of ER) was resuspended in 2 ml of SEM buffer containing 2 mM PMSF and homogenized with a dounce homogenizer. The sample was centrifuged (18,000 ***g***, 20 min, 4°C) to obtain ER/microsomes in the supernatant.

The obtained fractions were precipitated with 1:1 chloroform-methanol mixture and the pellet was resuspended in 2× sample buffer (125 mM Tris-HCl pH 6.8, 4% SDS, 20% glycerol, 10% β-mercaptoethanol, 2 mg/ml Bromophenol Blue) to obtain protein concentration of 2 mg/ml. Samples were heated at 95°C for 10 min and further analysed by SDS-PAGE and immunoblotting. Table S4 indicates the antibodies used in the current study.

#### Protein analysis

SDS-PAGE was used to separate protein samples. Samples were resuspended in 2× Laemmli buffer with 5% β-mercaptoethanol, boiled at 95°C for 10 min and loaded for analysis onto gels ranging from 10 to 15% acrylamide. Proteins were subsequently transferred onto nitrocellulose membranes and immunodecorated. Quantification of western blots was performed using the AIDA software.

To analyse proteins in their native state, BN-PAGE was used; isolated mitochondria were resuspended in 1% digitonin (digitonin to protein ratio of 6:1 for immunodecoration with antibodies against Tom40 or ratio of 1:1 for detection of assembled radiolabelled Tom20 molecules) in SEM buffer. After 30 min incubation, the samples were centrifuged (30,000 ***g***, 20 min, 2°C) and the supernatant was mixed with 10× BN loading dye (5% (w/v) Coomassie Brilliant Blue G-250, 100 mM Bis-Tris, 500 mM 6-aminocaproic acid, pH 7.0). After the separation on the gel, proteins were blotted onto polyvinylidene difluoride (PVDF) membrane and immunodetection proceeded as for SDS-PAGE. The raw data for all presented immunodecorations could be found in Fig. S2.

#### *In vitro* import assay

For the *in vitro* import assay with radiolabelled proteins and isolated mitochondria, a previously published protocol was used (Mokranjac and Neupert, 2007). In this study, a mixture of radiolabelled cysteine and methionine (Revvity, cat. #NEG772002MC) was used during the translation of the proteins. Accordingly, excess of unlabelled methionine and cysteine were used at the end of the translation step. The import of pSu9-DHFR was followed by proteinase K (PK, 50 µg/ml; Roche, cat. #3115887001) treatment and the activity of PK was inhibited using 5 mM PMSF. The import of Tom20–3xHA was followed by BN-PAGE. After import at 25°C for various time periods, the isolated mitochondria were collected by centrifugation (13,200 ***g***, 10 min, 2°C) and then resuspended in SEM buffer containing digitonin. Further analysis was as described above.

### Microscopy

To study mitochondrial morphology of yeast cells via fluorescent microscopy, yeast strains were transformed with the pYX122-pSu9-GFP plasmid resulting in GFP-stained mitochondria. Cells were grown to an OD_600_ of 0.8–1.0, harvested and mixed with 1% (w/v) low-melting-point agarose (Sigma, cat. #A9414) to limit their movement on the coverslip. Fluorescence images were taken using a confocal spinning disk microscope Zeiss Axio Examiner Z1 with a CSU-X1 real-time confocal system (Visitron) and SPOT Flex charge-coupled device camera. Fluorescence images were analysed using ImageJ (Fiji).

## Supplementary Material

10.1242/joces.263736_sup1 Supplementary information
