# Supplementary material for: The mammalian protein MTCH1 can function as an insertase
Source: J Cell Sci. 2025 Aug 15;138(16):jcs263736. doi: 10.1242/jcs.263736 (PMC12401536; doi:10.1242/jcs.263736)
Supplement: Supplementary information [file joces-138-263736-s1.pdf]

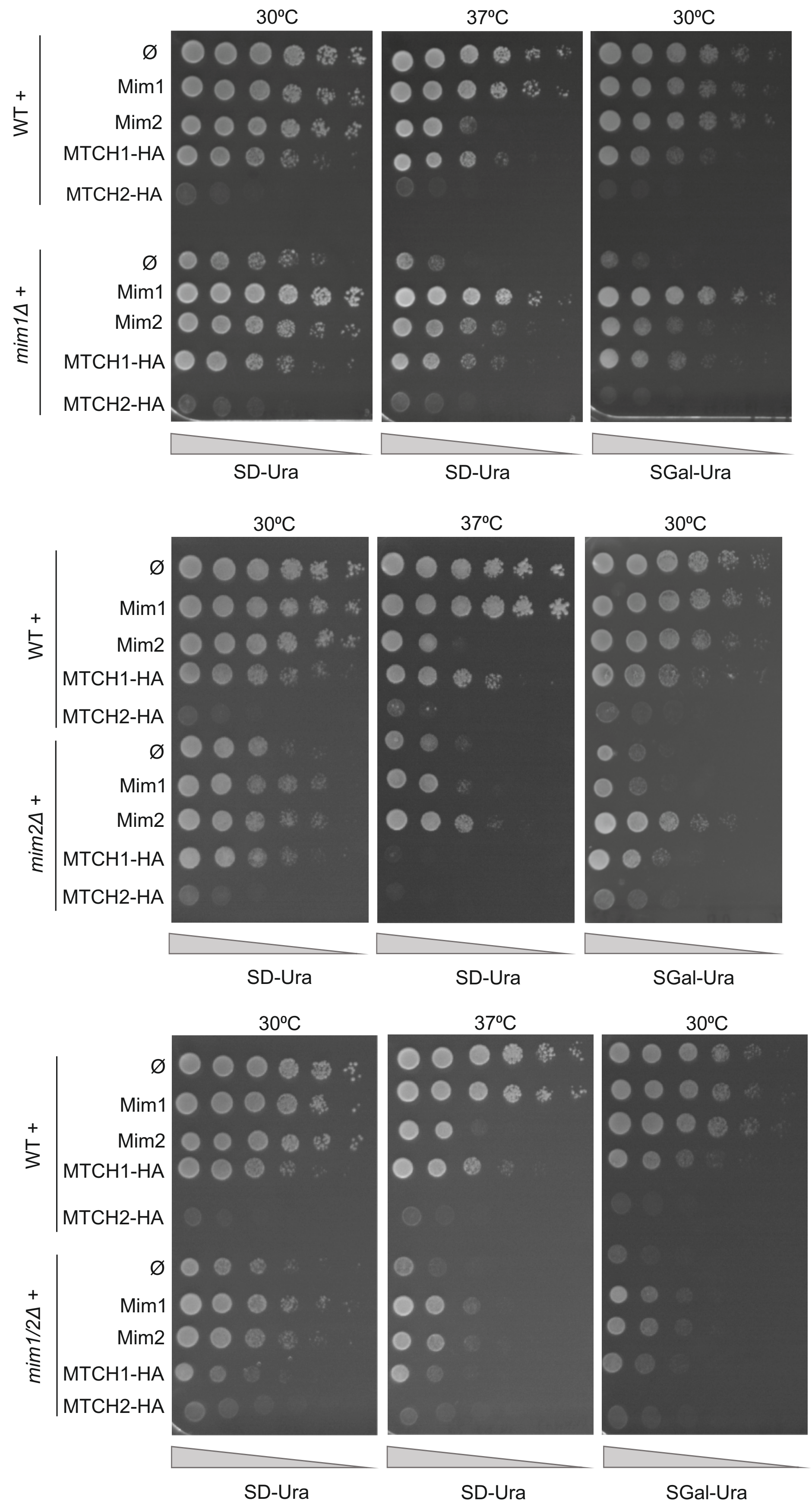

Fig. S1. HA-tagged MTCH1 can rescue growth retardation in cells mutated for Mim components. The growth of the indicated strains was monitored by drop dilution assay on solid synthetic medium containing either glucose (SD) or glycerol (SG) at either 30ºC or 37ºC. The strains were transformed with an empty vector (Ø) or vector encoding the indicated protein. Plates were incubated for 3 days at the indicated temperature before pictures were taken.

Journal of Cell Science • Supplementary information

Figure 3C.

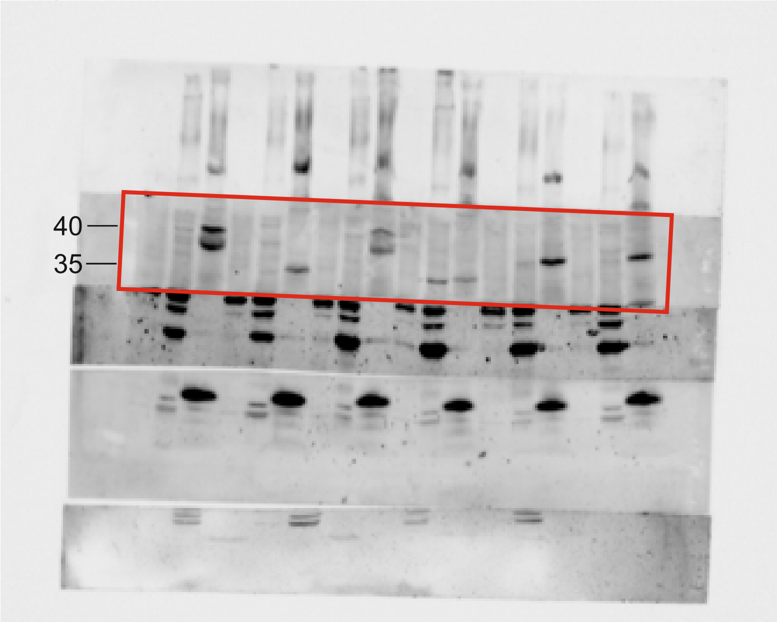

αHA

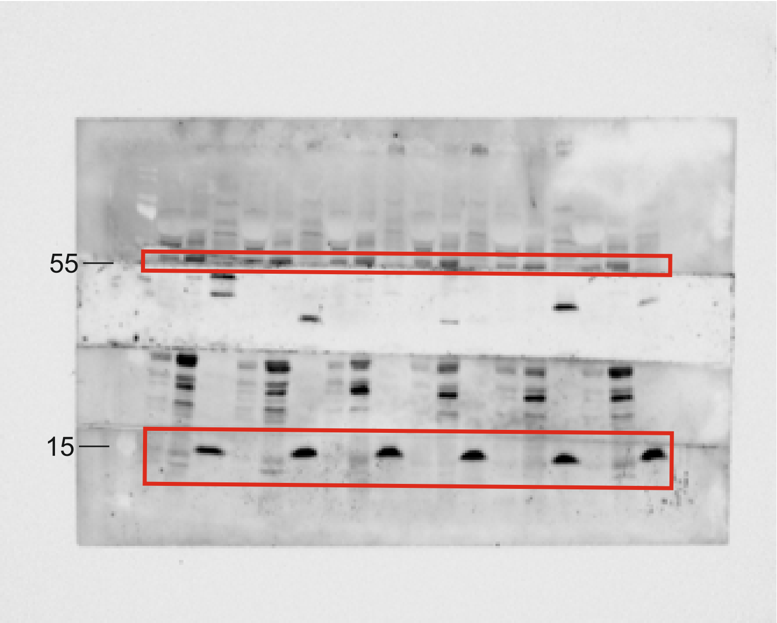

αHxk1

αHep1

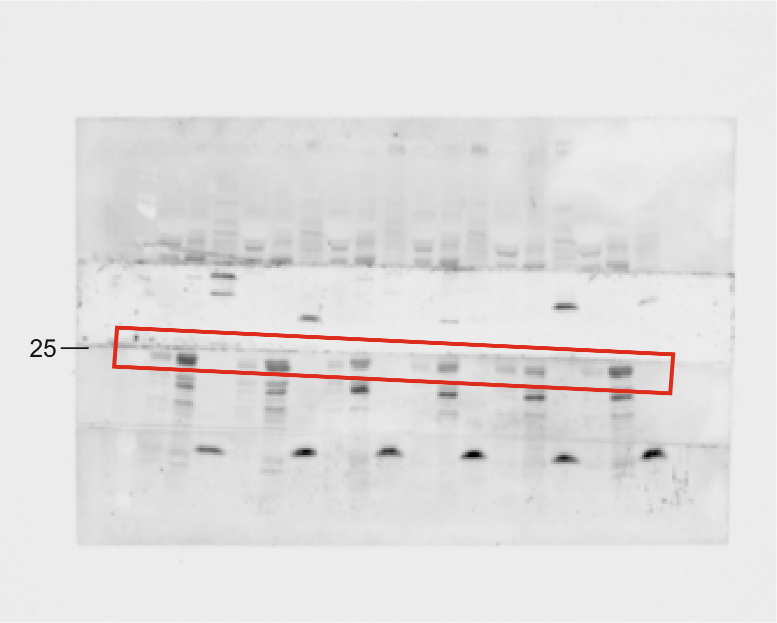

αErv2

Journal of Cell Science • Supplementary information

Figure 3D.

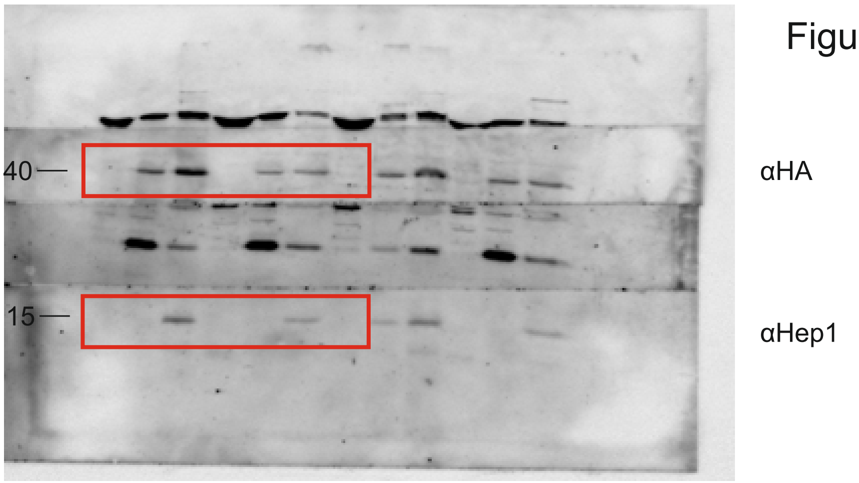

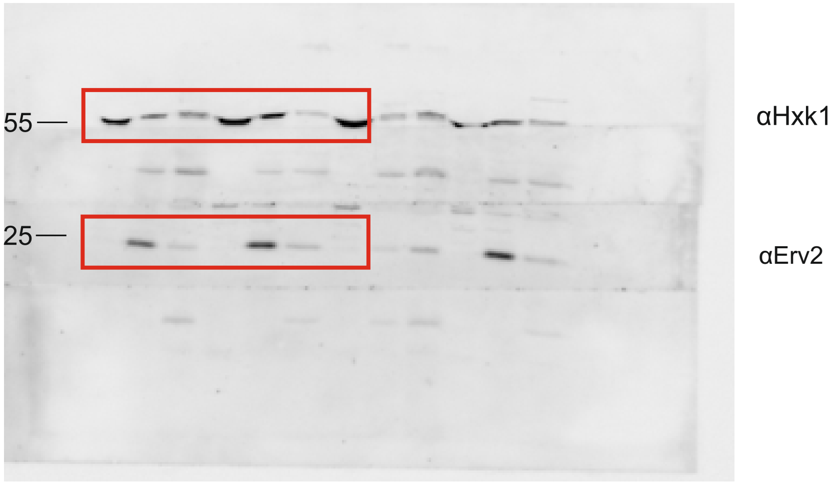

Figure 4A.

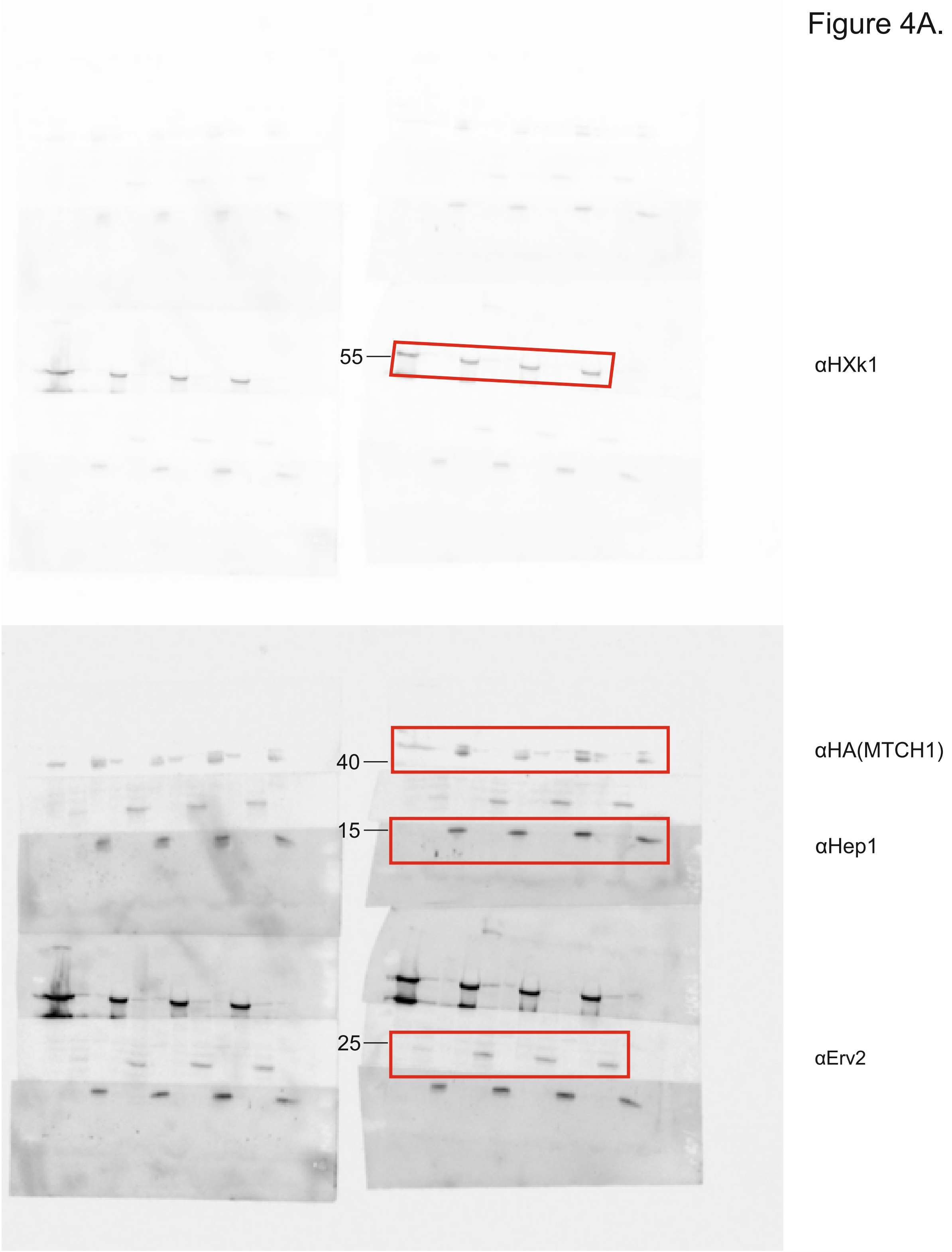

Journal of Cell Science • Supplementary information

Figure 4B.

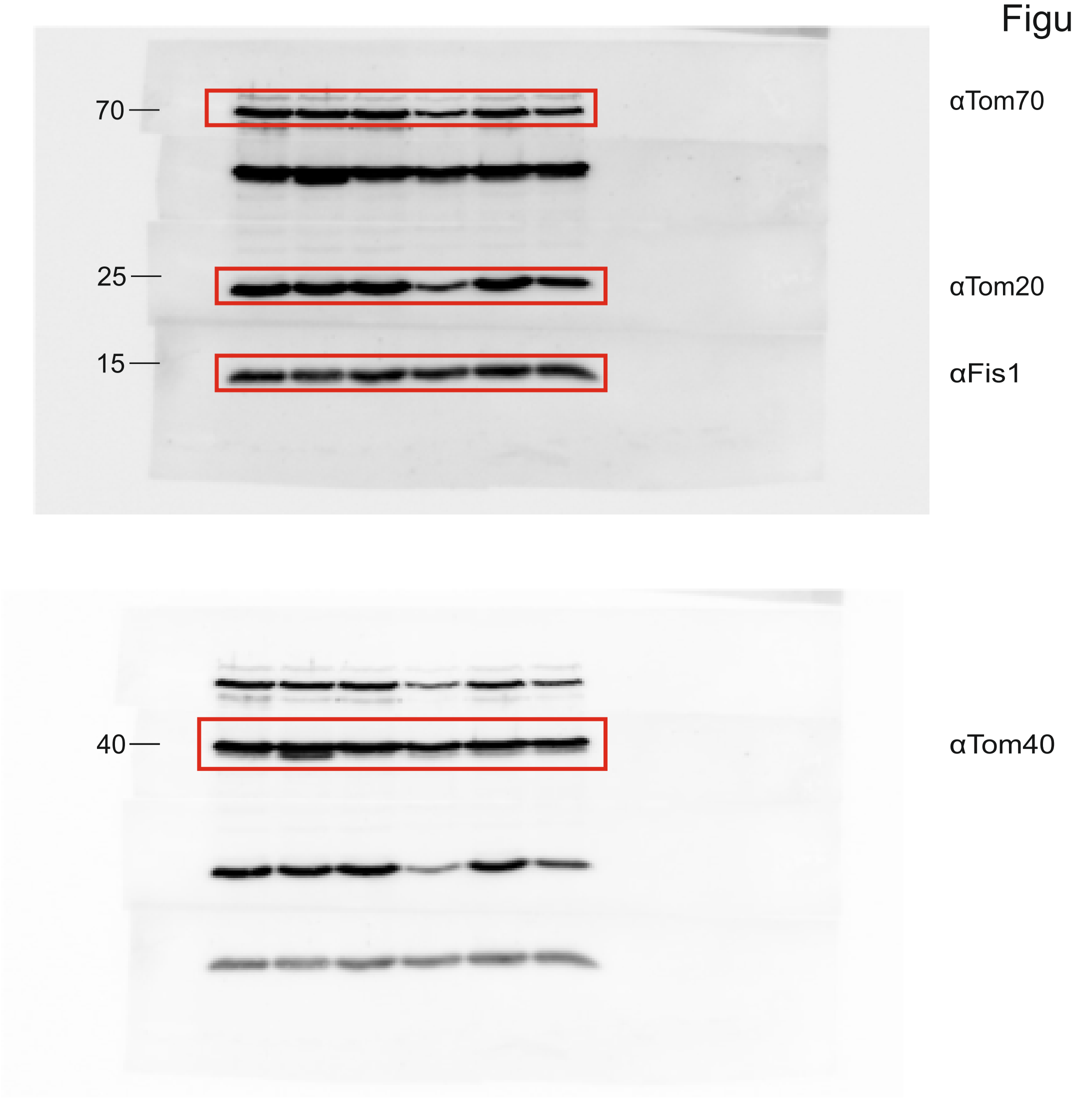

Figure 4D.

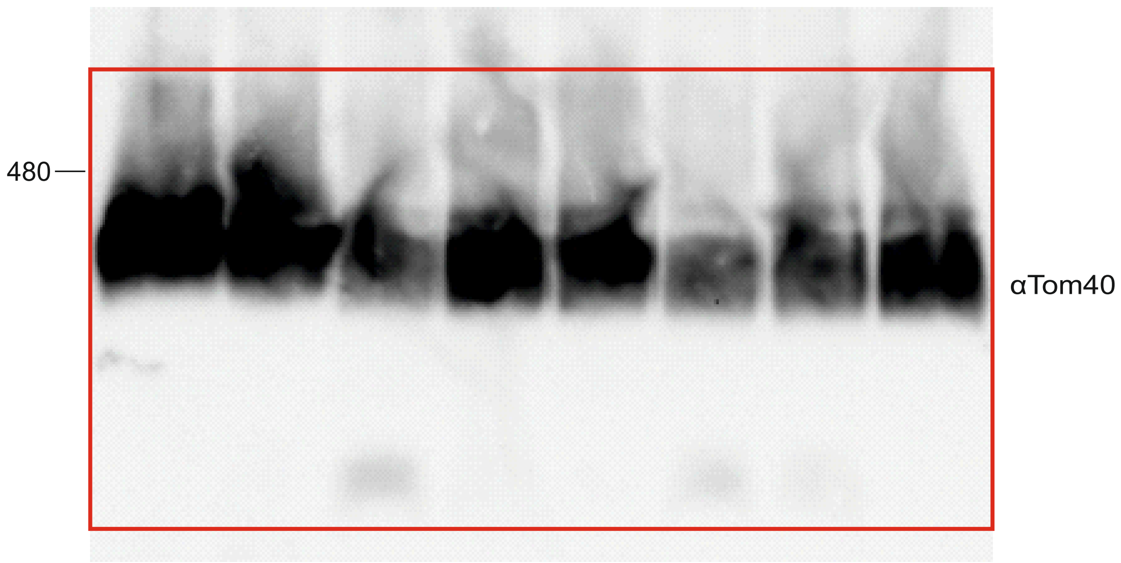

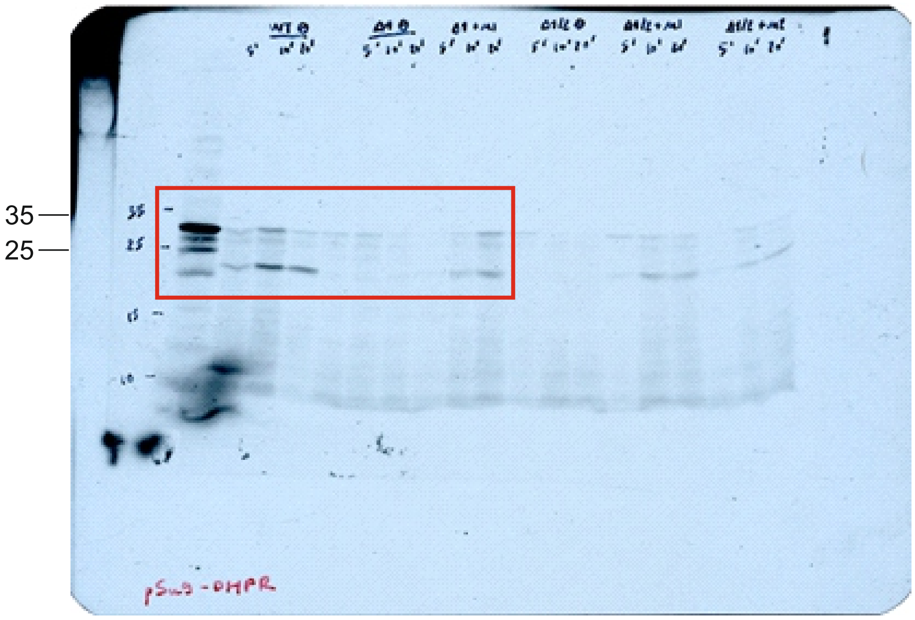

Figure 4E.

Figure 4E.

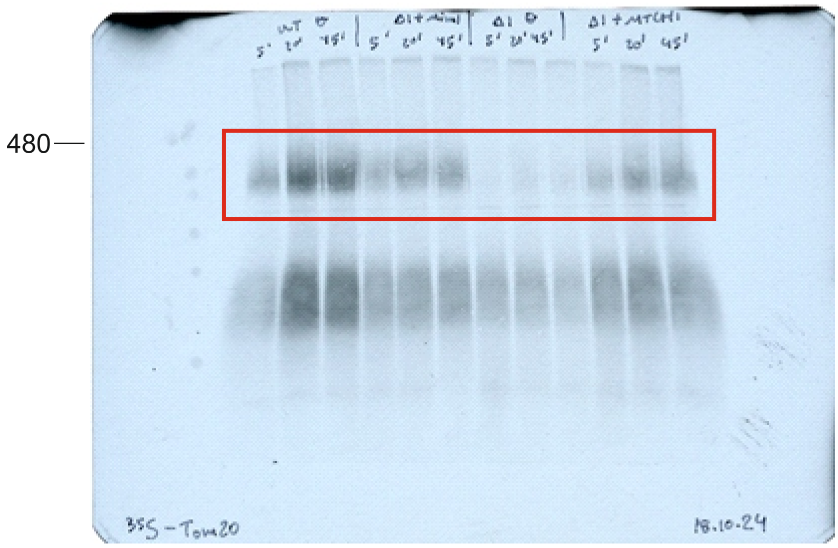

Fig. S2. Blot Transparency

Journal of Cell Science • Supplementary information

Table S1. Strains used in this study.

Strain	Genotype	Source or Reference
WT; W303α	N/A	DOI: 10.1242/jcs.103804
mim1Δ; W303α	MIM1::KanMX	DOI: 10.1242/jcs.103804
mim2Δ; W303α	MIM2::HIS3	DOI: 10.1242/jcs.103804
mim1Δ*mim2Δ*; W303α	MIM1::KanMX MIM2::HIS3	DOI: 10.1242/jcs.103804

Table S2. Primers used in this study.

Primer name	Sequence 5' to 3'	Note
MTCH1 yeast optimised F	AAGACACGAATTCATGGGTGCTTCTGATCCTG AAGTTGCC	Forward primer for yeast expression of MTCH1
MTCH1 yeast optimised R	AAGACACGGATCCTTATTCTAGCGCAAAACAG CTTCC	Reverse primer for yeast expression of MTCH1
MTCH2 yeast optimised F	AAGACACGAATTCATGGCAGATGCAGCTTCAC AAGTG	Forward primer for yeast expression of MTCH2
MTCH2 yeast optimised R	AAGACACGGATCCTTAGATAAGCATCTTTAAG TCGCA	Reverse primer for yeast expression of MTCH2
MTCH1-HA yeast optimised Rev primer	AAGACACGGATCCTTATGCGTAGTCAGGCACA TCATACGGATAAAGCTTTTCTAGCGCAAAACA GCTTCC	Reverse primer for yeast expression of MTCH1 with a C-terminal HA tag
MTCH2-HA yeast optimised R	AAGACACGGATCCTTATGCGTAGTCAGGCACA TCATACGGATAAAGCTTGATAAGCATCTTTAA GTCGCA	Reverse primer for yeast expression of MTCH2 with a C-terminal HA tag
MTCH1 no N F	AAGACACGAATTCATGGCACCAACTACGGAA GCCTTGTTT	Forward primer for yeast expression of MTCH1 without the N-terminus

The highlighting indicates the DNA sequence encoding the HA tag.

Table S3. Plasmids used in this study.

Plasmid	Promoter	Markers	Purpose	Source
pRS426	TPI	Amp^R, URA3	Yeast expression	Lab Stock
pRS426 Mim1	TPI	Amp^R, URA3	Yeast expression	This study
pRS426 Mim2	TPI	Amp^R, URA3	Yeast expression	This study
pRS426 MTCH1	TPI	Amp^R, URA3	Yeast expression	This study
pRS426 MTCH2	TPI	Amp^R, URA3	Yeast expression	This study
pRS426 MTCH1-HA	TPI	Amp^R, URA3	Yeast expression	This study
pRS426 MTCH2-HA	TPI	Amp^R, URA3	Yeast expression	This study
pYX142	TPI	Amp^R, LEU2	Yeast expression	Lab Stock
pYX142 Mim1	TPI	Amp^R, LEU2	Yeast expression	This study
pYX142 MTCH1-HA	TPI	Amp^R, LEU2	Yeast expression	This study
pYX142 MTCH2-HA	TPI	Amp^R, LEU2	Yeast expression	This study
pYX142 MTCH1(no N)-HA	TPI	Amp^R, LEU2	Yeast expression	This study
pYX142 MTCH1(N)+MTCH2-HA	TPI	Amp^R, LEU2	Yeast expression	This study
PYX122 pSu9-GFP	TPI	Amp^R, HIS3	Yeast expression	Lab stock
pGEM4 pSu9-DHFR	SP6/T7	Amp^R	In vitro transcription	Lab Stock
pGEM4 Om14	SP6/T7	Amp^R	In vitro transcription	Lab Stock
pGEM4 3xHA-Tom20	SP6/T7	Amp^R	In vitro transcription	Lab Stock
pSP6 PBR	SP6	Amp^R	In vitro transcription	Lab Stock
pEX-A258 MTCH1(N)+MTCH2-HA	lac	Amp^R	Subcloning	Eurofins

Journal of Cell Science • Supplementary information

Table S4. Antibodies used in this study.

Antibody	Source or reference	Dilution
Polyclonal rabbit αFis1	Lab Stock	1:1000
Polyclonal rabbit αTom20	Lab Stock	1:1000
Polyclonal rabbit αTom40	Lab Stock	1:1000
Polyclonal rabbit αTom70	Lab Stock	1:500
Polyclonal rabbit αErv2	Lab of Roland Lill	1:1000
Polyclonal rabbit αHep1	Lab Stock	1:1000
Polyclonal rabbit αHexokinase1	Bio-Trend (#100-4159)	1:1000
Polyclonal rat anti-HA	Roche (11867423001)	1:1000
Goat anti-rabbit IgG HRP conjugate	BioRad, 1721019	1:5000
